# New Appliances and Things Medical

**Published:** 1902-11-22

**Authors:** 


### NEW APPLIANCES AND THINGS MEDICAL.

[We shall be glad to receive, at our Office, 28 & 29 Southampton Street, Strand, London, W.C., from the manufacturers, specimens of all new preparations and appliances which may be brought out from time to time.]

### FERROLEUM.

(The Ferroleum Company, 86 Clerkenwell Road, London, E.C.)

This new preparation is a skilful combination of cod-liver oil, phosphate of iron, phosphorus, and glycerine, presented in the form of a fine emulsion, which, though tasting of cod-liver oil, is by no means so nauseating as the simple oil or many of the well-known emulsions which contain it. As a tonic, owing to the presence of phosphorus dissolved in oil, and of phosphate of iron dissolved in water and glycerine, ferroleum must be admitted to be of reliable and of great value, while the addition of cod-liver oil renders it a nerve food of undeniable merit. Ferroleum may usefully be tried in cases of nerve exhaustion and tissue loss, especially during convalesence from rheumatism.

### EUTHERMIC COVERLET.

(The Sanitary and Economic Association, Limited, Gloucester.)

The coverlet of the above name constitutes an exceedingly cheap and warm bed covering, and such as might well be supplied to infirm patients in our public institutions. The coverlet which consist of a case of cotton of artistic design is stuffed with a material called pinol, which is composed of a mixed fibre of vegetable origin, chiefly pine wool. The combination which is the result of numerous experiments made with various materials constitutes a most sanitary stuffing, elastic, porous, and comparatively light. There should, however, we think, be some provision for ventilation, as by small stitched holes. This would perhaps add to the expense, and since the object of using wood fibre instead of down, is to reduce the price so as to come within the reach of the poor, such ventilation might be reserved for coverlets of a slightly superior quality.

### THE IDEAL BROWN BREAD.

(Carr and Co., Limited, Millers, Carlisle.)

The brown bread made from Carr's celebrated malt meal possesses properties which give it a distinctive value. The malt meal itself contains nutritive elements which surpass those of ordinary whole meal, and the combination with malt renders them more easily digested and more rapidly assimilated. Bread made from this meal is delicious, wholesome, and appetising, and moreover it will keep fresh and moist for a much longer period than is the case with ordinary white bread.

